# Research on high-speed classification and location algorithm for logistics parcels based on a monocular camera

**DOI:** 10.1038/s41598-024-66941-x

**Published:** 2024-07-10

**Authors:** Zhehao Lu, Ning Dai, Xudong Hu, Kaixin Xu, Yanhong Yuan

**Affiliations:** Key Laboratory of Modern Textile Machinery&Technology of Zhejiang Province, Zhejiang Sci-Tech University, Hangzhou, 310018 China

**Keywords:** Logistics parcel positioning, Monocular camera algorithm, YOLOv5, High-speed logistics application, Engineering, Electrical and electronic engineering, Mechanical engineering

## Abstract

The rapid development of the logistics industry has driven innovations in parcel sorting technology, among which the swift and precise positioning and classification of parcels have become key to enhancing the performance of logistics systems. This study aims to address the limitations of traditional light curtain positioning methods in logistics sorting workshops amidst high-speed upgrades. This paper proposes a high-speed classification and location algorithm for logistics parcels utilizing a monocular camera. The algorithm combines traditional visual processing methods with an enhanced version of the lightweight YOLOv5 object detection algorithm, achieving high-speed, high-precision parcel positioning. Through the adjustment of the network structure and the incorporation of new feature extraction modules and ECIOU loss functions, the model’s robustness and detection accuracy have been significantly improved. Experimental results demonstrate that this model exhibits outstanding performance on a customized logistics parcel dataset, notably enhancing the model's parameter efficiency and computational speed, thereby offering an effective solution for industrial applications in high-speed logistics supply.

## Introduction

**Figure 1:**
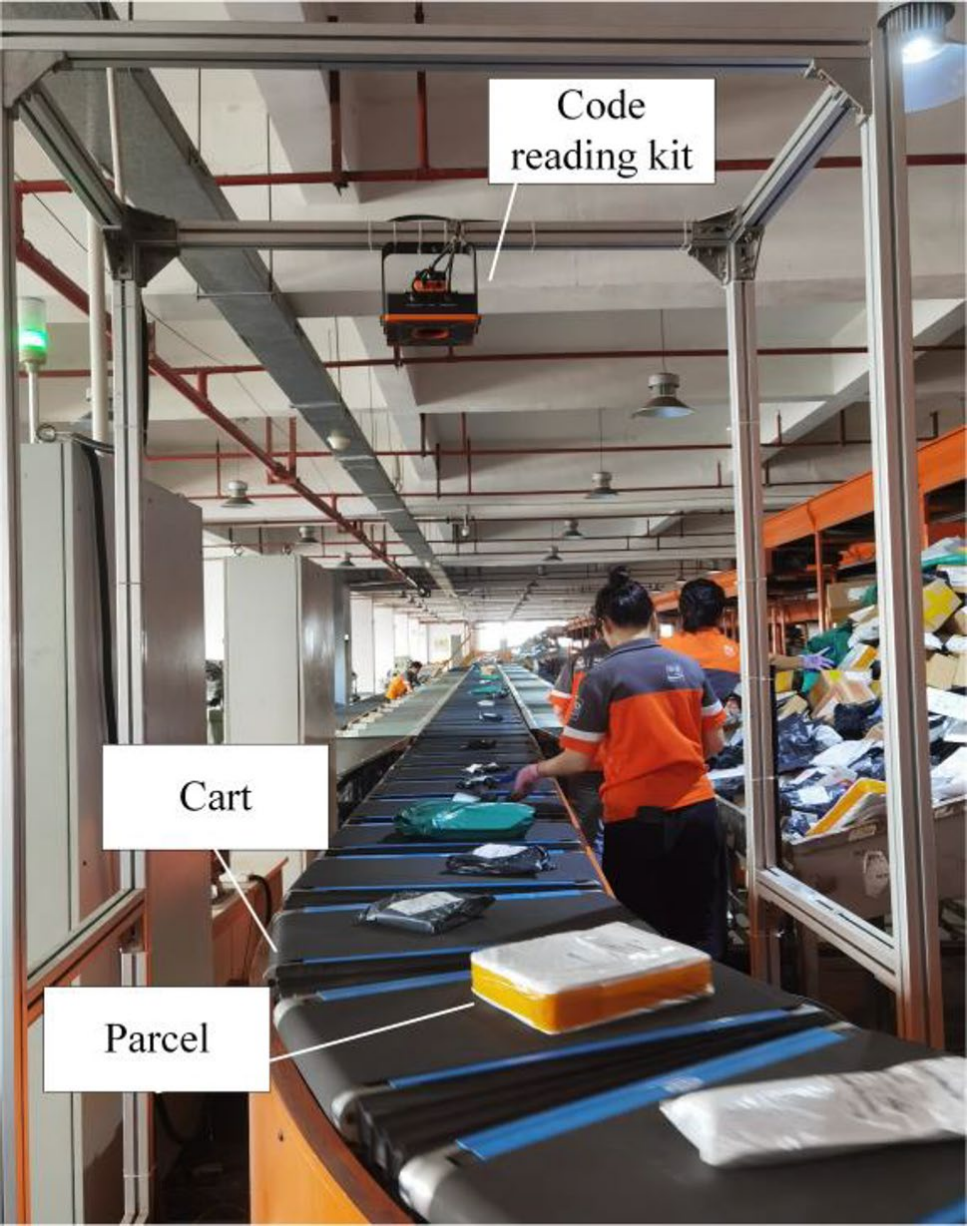
Cross-belt sorter.

The express delivery industry is an important sector in China's economic development, contributing efficient logistics services to various industries. Especially with the advent of the internet and e-commerce, the express industry has witnessed rapid development opportunities^1–4^. Owing to low labor costs, the majority of express delivery points still employ manual sorting. With the dramatic increase in parcel volume, these traditional manual sorting methods have demonstrated inefficiencies and high error rates, unable to satisfy market and customer demands^5–9^. To address this issue, the express industry has begun introducing various automated sorting equipment^10^, including airflow sorters, chute sorters, cross-belt sorters, and swing wheel sorters. Among these, the cross-belt sorter^11^ represents a type of advanced sorting equipment employing linear motors and other power units to connect carts end-to-end. As depicted in Fig. 1, the cart within the equipment rapidly traverses the track. Upon entering the code reading area, the code reading apparatus scans the barcode on the parcel, thereby acquiring the parcel’s destination grid information. Concurrently, each cart is equipped with a conveyor belt powered independently, enabling vertical movement in alignment with the cart's trajectory. This mechanism facilitates the seamless sorting of parcels into designated slots, thus accomplishing the automated sorting process. This system necessitates manual intervention to position parcels centrally on each cart, a requirement that limits operational speeds to levels manageable only by highly skilled workers.

**Figure 2:**
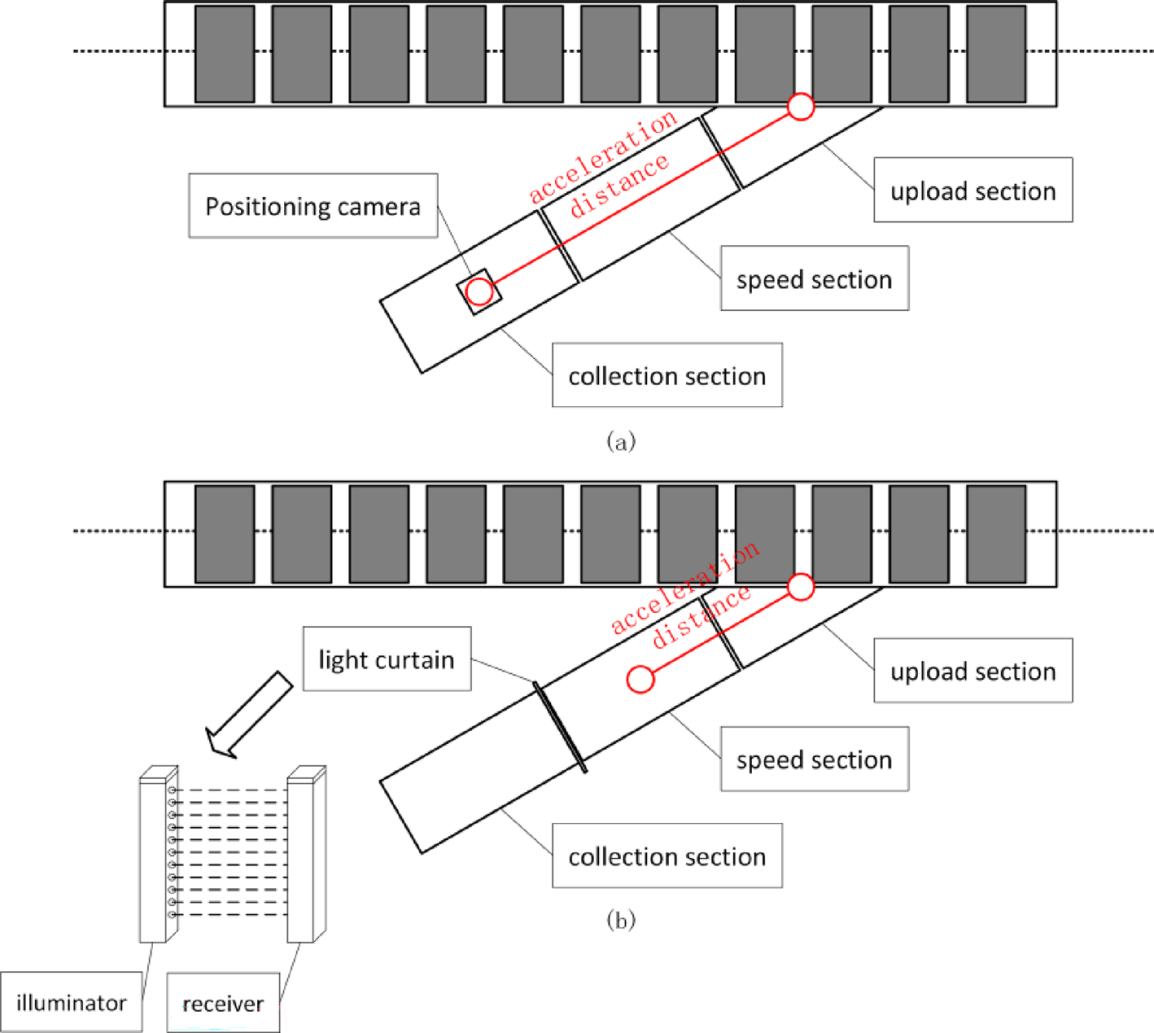
Schematic diagram of the parcel feeding station. (**a**) Monocular camera positioning. (**b**) Light curtain positioning.

Consequently, within the architecture of the cross-belt sorter, the parcel feeding station emerges as a pivotal piece of equipment. tasked with transferring parcels from the collection segment to the sorting carts, while also performing scanning, weighing, and positioning of the parcels. The feeding station is typically comprised of three parts: the collection segment, the speed adjustment segment, and the loading segment, as depicted in Fig. 2.

The collection segment incorporates a code reading and weighing system to obtain the barcode and weight information of the parcels. Utilizing this information, it identifies the drop-off slot and allocates the sorting cart. At the termination of the collection segment, a light curtain is positioned, the principle of light curtain positioning is that when an object passes through the light curtain, it blocks part of the light from the emitter, preventing it from reaching the corresponding receiver. Using synchronous scanning technology, it becomes possible to identify which channels are blocked and which are open. The size and position of the object can be determined based on the number, duration, and other details of the blocked beams. This method entails calculating the parcel's width from the number of blocked beams and deriving the length from the product of the belt speed and the duration of blockage. Simultaneously, the parcel’s central position is determined based on the number of blocked beams. Upon traversing the light curtain, the parcel halts at the center of the speed adjustment segment, anticipating the arrival of the assigned cart. The loading speed of the parcel onto the cart is ascertained based on the center point and weight information of the parcel. This guarantees that the speed of the collection segment aligns with the speed of the loading segment’s conveyor belt upon reaching the loading segment, thereby preventing slippage. The loading segment employs a narrow belt conveyor, with a transport speed that is consistent with the loop line’s speed, enabling the accurate placement of parcels into the center of the high-speed moving sorting cart’s conveyor belt, thereby concluding the feeding process.

**Figure 3:**
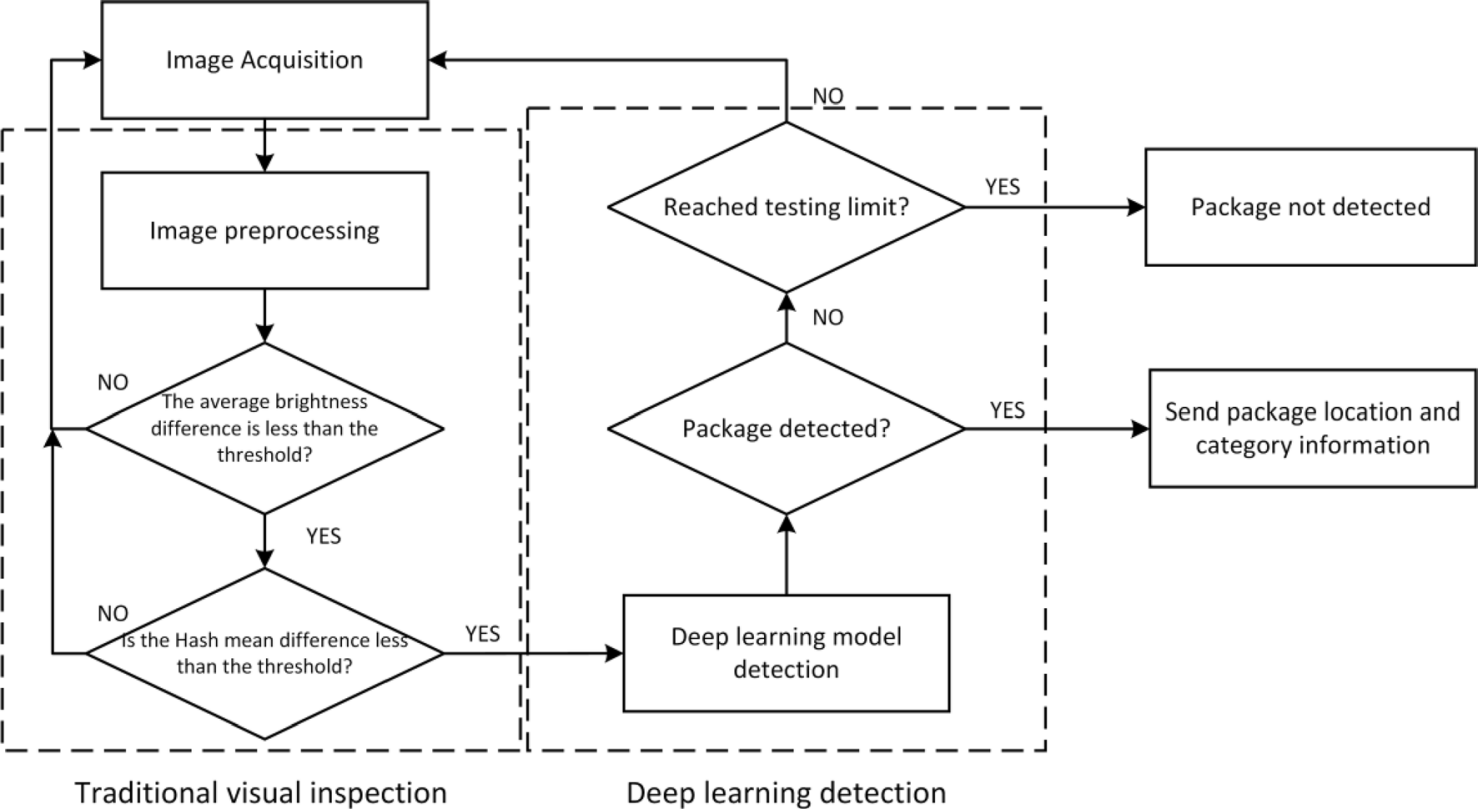
Parcel classification and positioning algorithm.

While this traditional light curtain positioning method is straightforward and user-friendly, it presents several limitations and drawbacks. First, it necessitates high stability of the belt speed; fluctuations can compromise the accuracy of length information. Second, it exhibits limited adaptability to the installation environment; the logistics workshop, rife with dust and unavoidable bumps, renders the system prone to dust accumulation and damage. Third, its measurement error equals twice the distance between each pair of sensors. Achieving higher accuracy requires additional infrared transmitters and receivers, substantially elevating costs and impacting equipment stability. Furthermore, as the parcel must halt in the middle of the speed adjustment segment post-light curtain traversal, without altering the mechanical structure, its acceleration distance frequently fails to fulfill the requirements for high-speed parcel feeding, illustrated in Fig. 2(b). Consequently, to satisfy the acceleration distance requirements for high-speed parcel feeding, enhance the accuracy and adaptability of parcel positioning, and diminish costs, the implementation of more advanced technologies and methods, like image processing and deep learning, is essential. Addressing these limitations, this paper introduces an intelligent classification and location algorithm for logistics parcels utilizing a monocular camera. The overall framework of this algorithm is depicted in Fig. 3.

The contributions of this study include:

1. Acknowledging the diverse friction attributes of various parcels, this research has compiled a novel dataset for classifying parcels. This dataset is capable of distinguishing between different parcel types, significantly mitigating the effects of variance in parcel friction coefficients.
2. The paper proposes a cutting-edge framework for parcel identification that synthesizes traditional vision techniques with deep learning methodologies. This framework is designed to reduce manual intervention to the bare minimum while maximizing the precision of parcel identification.
3. Tailoring to the distinct characteristics of parcels, an enhanced version of the YOLOv5 model has been developed for the purpose of detection. The empirical outcomes affirm the model’s capability to detect and classify parcels with high efficiency and accuracy.

## Related work

In the logistics industry, vision systems have emerged as a key technology to enhance automation levels and operational efficiency. Initially, these cameras were used primarily to perform simple tasks such as barcode scanning and basic image recognition, utilizing traditional image processing techniques such as edge detection and color segmentation. Ladplee et al.^12^ proposed an automated system for the volume measurement of rectangular parcel boxes using a single LiDAR depth camera. The system employs a flood filling algorithm to detect the top surface and enhances height accuracy via grid sampling, achieving a low measurement error of 5% and a processing time of approximately 1 s. Monica et al.^13^ designed a parcel detection system using a 3D camera that can identify cardboard boxes of known size on a pallet through edge detection and other techniques. Among various vision systems, monocular cameras are widely utilized in the logistics industry as a cost-effective option. Yunardi et al.^14^ developed a contour-based object detection system for automated sorting, measuring parcel boxes using 2D images captured by two monocular cameras for pixel calculation and calibration comparisons, achieving a detection rate of 87.5%. However, while these techniques are effective, they often face limitations due to lighting conditions and image quality, complicating their application in the dynamic and complex logistics environments.

Over time, the advancement of deep learning technology has significantly enhanced the innovation of vision systems in logistics parcel processing. The adoption of deep learning, particularly convolutional neural networks (CNN), offers powerful tools for processing complex images, enabling automatic feature learning that distinguishes parcels from extensive datasets without manual feature design. This capability markedly improves recognition accuracy and robustness under conditions of variable illumination, diverse backgrounds, and varied parcel shapes and sizes. Han et al.^15^ utilized a visual parcel sorting method based on multi-task deep learning, developed a lightweight object detection network model, and proposed an optimal sorting position and orientation based on key points. The estimated multi-task model achieved a sorting success rate of 89.7% at an inspection speed of 50 FPS. Zhao et al.^16^ developed an improved parcel detection model based on Faster R-CNN, employed a Sobel filter to construct an edge detection branch, and designed a self-attention ROI alignment module, achieving a detection accuracy of 98.76%. Zhang et al.^17^ proposed a two-level lightweight deep learning model named SFYOLOv5 for logistics parcel detection. The model utilizes Pruned-Shuffle-Block (PSB) and Focus for Downsampling (FFD) modules to reduce the number of operations and parameters involved in feature extraction. Experimental results indicate that the model achieved an average accuracy of 99.1% on the self-built logistics parcel data set. Vismanis et al.^18^ developed a robotic system for post office parcel processing. The system incorporated a deep learning-based RGB-depth sensor and demonstrated its efficient parcel processing capabilities through tests in five diverse scenarios. It is well-suited to handling complex post office parcel scenarios. Xu et al.^19^ proposed a logistics parcel identification method based on deep learning and multi-information fusion, integrating the detection and identification of barcode and three-segment code information, utilizing the enhanced YOLOv4 network^20^ for target detection, and employing ZBAR algorithm and Tesseract-OCR technology for recognition, achieving a recognition accuracy of 98.5%, which is more robust and accurate than traditional single barcode recognition methods.Wu et al.^21^ proposed an enhanced Faster RCNN framework integrating the adaptive Mosaic method to address parcel dataset imbalance and utilized lightweight networks to boost performance under reduced hardware requirements, ultimately decreasing the model’s parameters by 3.07 M. Kim et al.^22^ employed 3D machine vision and RGB-D imaging technologies to detect and identify goods, incorporating deep learning algorithms to enhance the precision of parcel boundary detection, thereby ensuring high efficiency and accuracy in parcel identification. Zhao et al.^23^ proposed a 3D-OAS model, augmented with a graph neural network, to markedly enhance both the efficiency and accuracy of parcel picking in complex stacking environments. Feng et al.^24^ developed a logistics sorting algorithm based on deep learning and empirical evaluation, focusing on the generation of positioning and grasping points for intelligent robots in logistics sorting. Utilizing advanced YOLOv3 target detection and instance segmentation technologies, they achieved a detection accuracy of 94.05%. Although deep learning technology is extensively applied in logistics parcel detection scenarios, current research has yet to fully classify logistics parcels or achieve optimal benchmarks in detection accuracy and speed.

## Materials and methods

### Data collection

**Figure 4:**
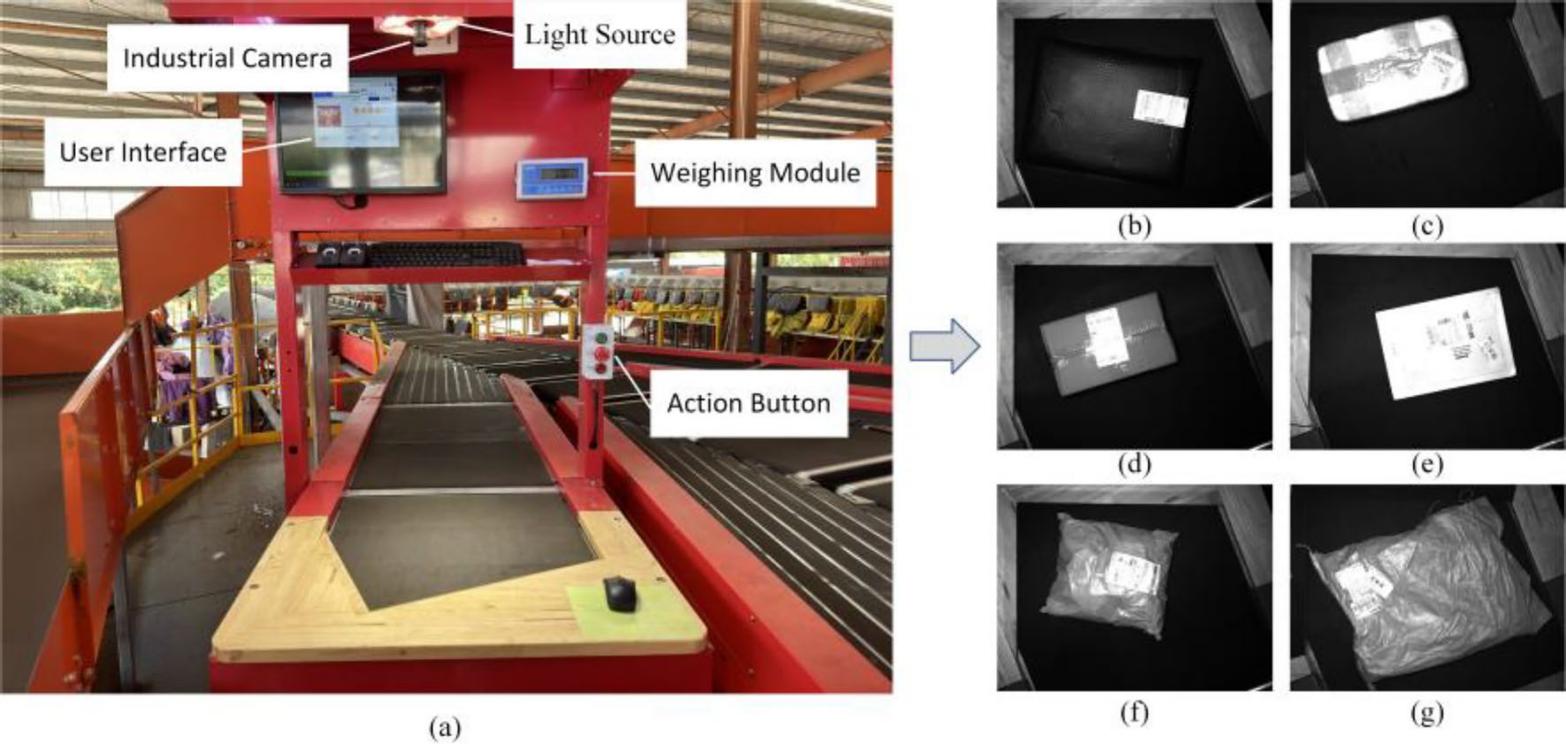
Schematic diagram of image acquisition structure.

Owing to the limited availability of public datasets for logistics parcel target detection, this study employed a self-built logistics parcel dataset. The dataset was gathered from a logistics site in Lishui City, Zhejiang Province, ensuring privacy protection during the collection process and being utilized exclusively for scientific research. The real-life images, as depicted in Fig. 4(a), were captured using Daheng Imaging’s MER2-1220-32U3M industrial camera, featuring an image pixel size of 4024 × 3036.

**Figure 5:**
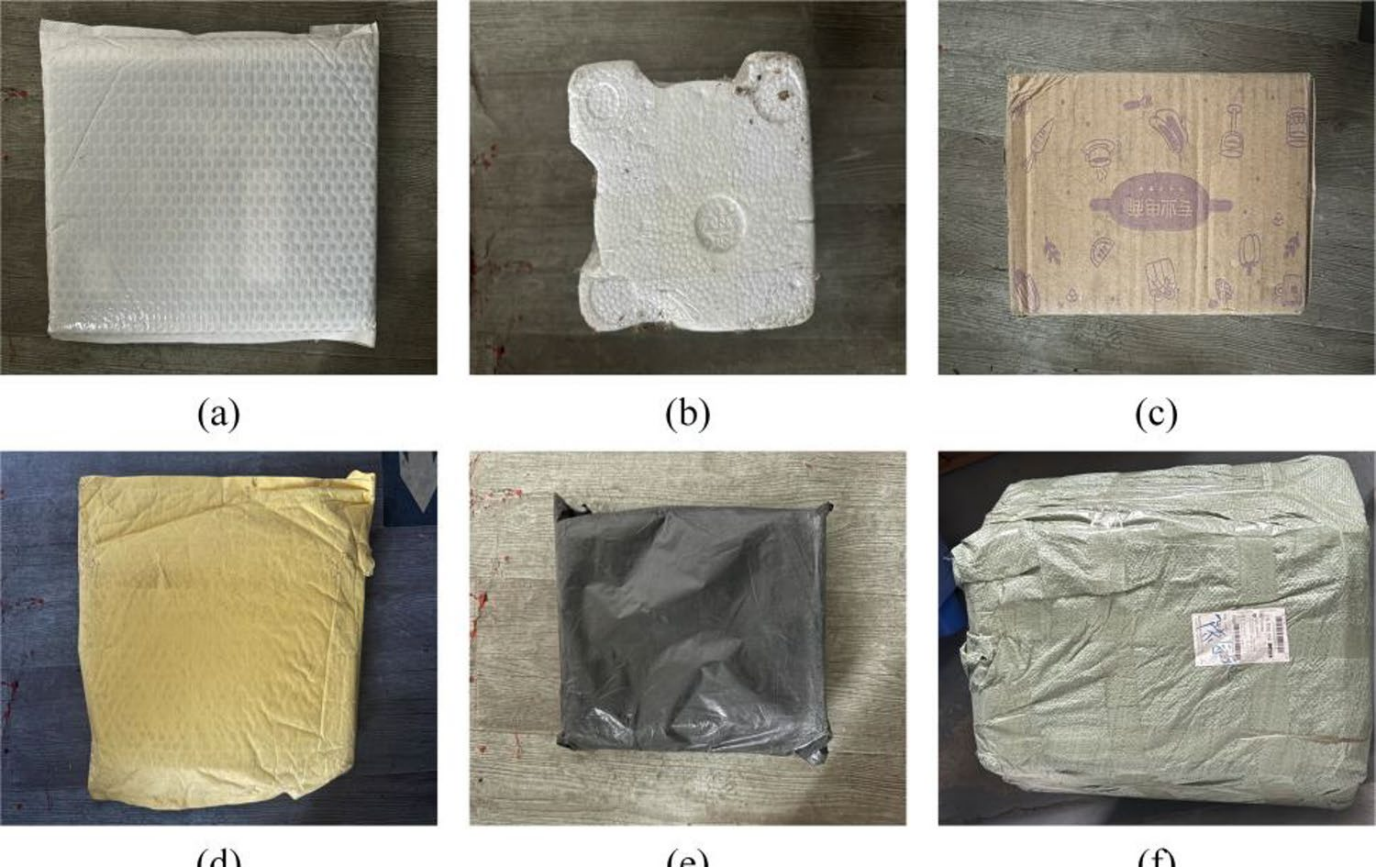
Physical pictures of six types of parcels.

As the parcel enters the sorting cart from the loading section, navigates curves, and exits, its movement—driven by the conveyor belt—results in variable friction coefficients. The significant influence of friction variation is evident as parcels with low friction coefficients slip more easily when the belt stops. This discrepancy from the expected displacement distance leads to sorting failures, a problem particularly pronounced in high-speed sorting scenarios. To mitigate this impact and decrease the parcel error rate (the probability that a parcel does not land in the designated grid), we categorize parcels into six types: bubble, foam, box, paper, plastic bag, and woven bag, as illustrated in Fig. 4(b)–(g) and Figs. 5(a)–(f). Woven bags, typically made of materials like polypropylene, have the roughest surface and consequently the highest friction coefficient. Foam materials, mainly consisting of foam plastic, also have relatively rough surfaces and follow closely in friction coefficient. Boxes are third, due to their friction-enhancing textures. Next are bubble; although these materials are somewhat rough, their friction coefficients are lower than the first three types. Paper materials, with their relatively smooth surfaces, rank fifth. Plastic bags, the smoothest, therefore, have the lowest friction coefficient. After classifying the parcels, we can halt the conveyor belt at different positions based on the average slip distance of each parcel type. Parcels that are more prone to slipping will be stopped earlier, allowing them to better align with our expected movement trajectory. Finally, we selected a subset of the images from the collected dataset, comprising 3625 images, and the number of various types of parcels is shown in Table 1. After annotating the images with LabelImg, we randomly divided them into training, verification, and test sets in a ratio of approximately 4:1:1.

**Table 1:** Statistics table of quantity of different parcels.

Parcel type	quantity
Bubble	719
Foam	217
Box	1093
Paper	383
Plastic	1056
Woven	157

**Figure 6:**
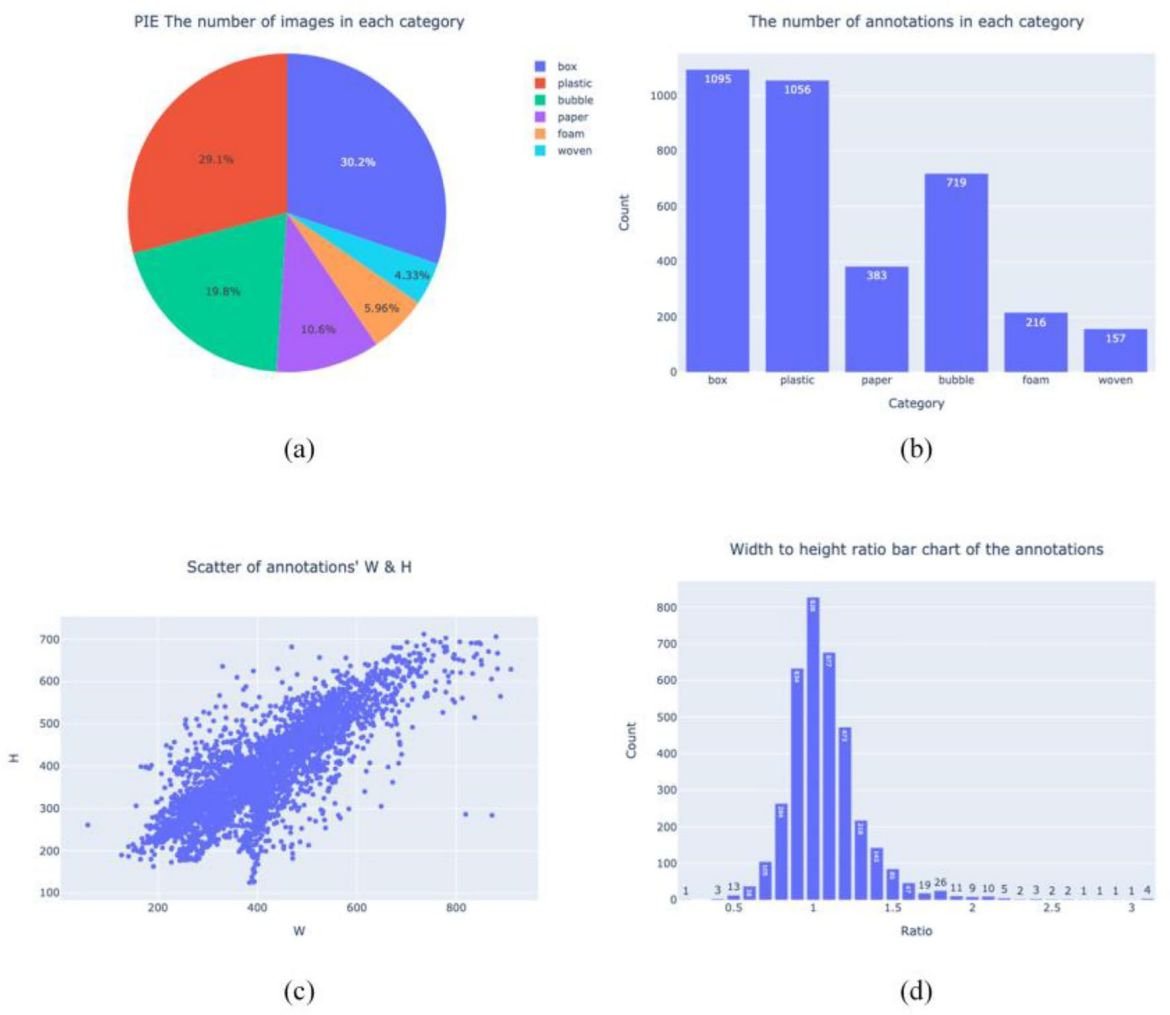
Parcel dataset feature analysis. (**a**) PIE the number of images in each category. (**b**) The number of annotations in each category. (**c**) Scatter of annotations’ W & H. (**d**) Width to height ratio bar chart of the annotations.

We analyzed this parcel dataset and identified several key characteristics: Firstly, as indicated by Table 1 and Fig. 6(a)(b), the distribution of parcels across categories is uneven. The “box” and “plastic” categories contain the highest proportions of images, approximately 30.2% and 29.1% respectively, while the “foam” and “braid” categories have significantly lower proportions, accounting for only 4.33% and 5.96% respectively. Secondly, as demonstrated in Fig. 6(c), within an image resolution of 1006 × 759 pixels, the dimensions of most parcels range between 200 and 600 pixels, indicating that the targets predominantly consist of larger items. Lastly, as shown in Fig. 6(d), although the aspect ratio of most parcels is concentrated between 1 and 1.5, there are also parcels with extreme aspect ratios. These particularly slender parcels require special consideration during identification.

### Traditional visual filtering

#### Filtering of human hands

**Figure 7:**
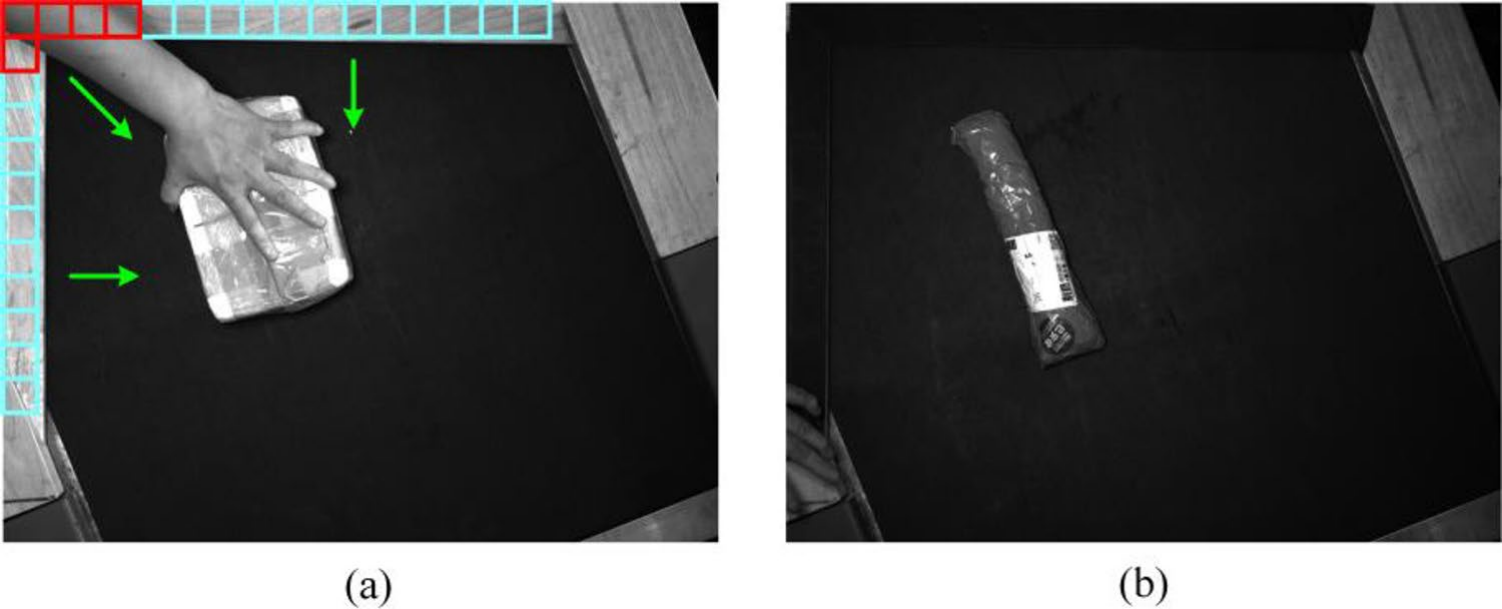
Schematic diagram of traditional visual filtering.

In the automatic parcel detection system, the operator's hands frequently enter the detection area, as depicted in Fig. 7(a). This not only compromises the accuracy of parcel positioning but also indicates that the position of the parcel might still be adjustable, presenting a potential for change. To effectively eliminate interference caused by the presence of hands, this study employs a filtering algorithm based on the average gray value.

As depicted in Fig. 4(a), the action of workers placing parcels onto the equipment typically takes place within an area demarcated by two right-angle boundaries. For a detailed analysis, this area is subdivided into A horizontal and B vertical segments. Within each segment, the algorithm computes the average gray value and compares it to a predefined normal value. The formula for calculating the average gray value is as follows:

1 $$\overline{G} = \frac{1}{mn}\sum\nolimits_{i = 1}^{m} {\sum\nolimits_{j = 1}^{n} {g(i,j)} }$$

where $m$,$n$ are the length and width of the image, and $g(i,j)$ is the grayscale value of the image at position $(i,j)$.

If the difference between the average gray value of a segment and the normal value exceeds a preset threshold, that segment is marked as containing interference. By tallying the segments that surpass this threshold, we can ascertain whether the operator's hand remains within the detection area, thereby effectively filtering out interference.

This method not only enhances detection accuracy but also optimizes processing speed by focusing solely on potential interference segments rather than the entire image.

#### Parcel stationarity determination

In automated parcel handling systems, confirming the stabilization of a parcel is a critical step for ensuring operational accuracy. Particularly for parcels with uneven bottoms, they may remain unstable even when placed on a flat surface due to their irregular bottom structures, as depicted in Fig. 7(b). To accurately determine the resting state of a parcel, this study employs the average hashing algorithm to analyze changes between two consecutive image frames.

The average hashing algorithm initiates by converting the input image to grayscale and then resizing it to a uniformly small dimension, simplifying the processing complexity. It calculates the grayscale average of all pixels in the resized image. Each pixel’s grayscale value is then compared with this average: pixels with values above the average are assigned a corresponding hash bit ($HashBit$) set to 1, and those at or below the average are set to 0, thus generating a 64-bit binary hash string ($Hash\_Code$) that represents the image.

2 $$HashBit = \left\{ \begin{gathered} {}^{^{\prime\prime}}1^{^{\prime\prime}} ,ifg(i,j) > \overline{G} \hfill \\ {}^{^{\prime\prime}}0^{^{\prime\prime}} ,otherwise \hfill \\ \end{gathered} \right.$$

To quantify the difference between consecutive image frames, the Hamming distance $H$ between two hash strings is calculated. This distance represents the number of positions at which the corresponding bits are different. By comparing this value with a preset minimum fluctuation threshold, a Hamming distance that is less than or equal to the threshold indicates that the parcel is in a stationary state. Conversely, a distance greater than the threshold suggests that the parcel may still be moving or shaking.

3 $$H = \sum\nolimits_{i = 1}^{n} {(Hash\_Code1[i] \ne Hash\_code2[i])}$$

By implementing this algorithm, we significantly enhance the response speed and accuracy during parcel processing, ensuring the accuracy and stability of subsequent automated operations. Moreover, this method provides a reliable technical means for the precise identification of dynamic and static targets within automated parcel processing systems.

### Deep learning detection

#### Baseline

YOLOv5 was employed as the parcel positioning algorithm. Representing an extension of the YOLO series^25^, YOLOv5 builds upon improvements made in YOLOv3^26^ and YOLOv4. In contrast to previous models, YOLOv5 enhances detection accuracy while preserving detection speed. It features five structures: YOLOv5n, YOLOv5s, YOLOv5m, YOLOv5l, and YOLOv5x, with the primary differences lying in the number of convolution kernels and specific part bottlenecks. The parameters of these five models escalate sequentially, as do their detection accuracies, but their detection speeds diminish in correlation. The inference speeds of each model are depicted in Table 2. In real-world parcel sorting environments, rapid recognition speed is imperative given limited hardware capabilities, imposing stringent model size limitations and prompting the selection of the YOLOv5n model for experimentation.

**Table 2:** YOLOv5 model detection speed test table.

Model	FPS
Yolov5n	84.7
Yolov5s	74.2
Yolov5m	64
Yolov5l	51.5
Yolov5x	42.3

#### Improved network structure

Observation of the label distribution within the logistics parcel dataset reveals that the recognition targets are predominantly large, often characterized by complex internal structures and rich semantic information. The identification of these large targets relies on deep features that are capable of capturing their overall shape and structure. In contrast to shallow features, which primarily provide surface texture information, deep features are prioritized for their superior ability to convey semantic information. This distinction becomes particularly significant in parcel supply scenarios with optimal lighting conditions and high contrast. In such environments, shallow texture features exhibit low discriminatory power and are minimally affected by variations in lighting and contrast. Consequently, deep semantic features become crucial, given their efficacy in capturing the comprehensive morphology and structure of the target, which is essential for precise target detection under favorable lighting conditions.

Furthermore, although the hand filtering method based on traditional vision is employed, it may inevitably fail under complex ambient lighting conditions, leading to the logistics parcel being obscured by the operator's hands. In scenarios of partial occlusion, deep semantic features demonstrate their efficacy in addressing target occlusion issues, owing to their robust context understanding capabilities. This advantage enables the model to more accurately infer the integrity of the target.

**Figure 8:**
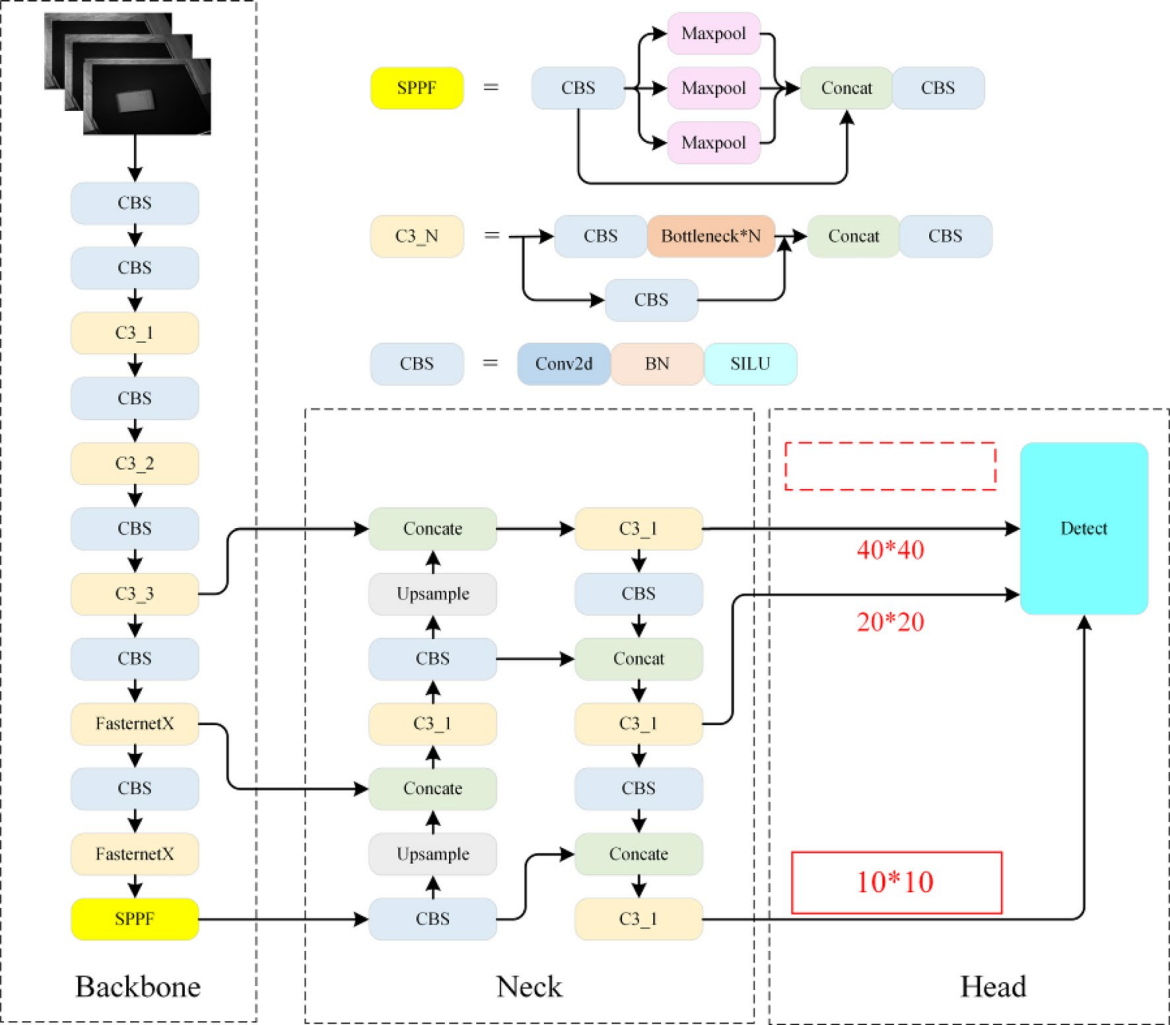
Improved network structure.

Based on the considerations outlined, significant modifications were made to the original YOLOv5 network structure, as illustrated in Fig. 8. These enhancements predominantly target the backbone and neck sections of the network to accommodate the addition of a newly designed 10 × 10 detection head, specifically tailored for capturing deep semantic information of large targets. In the backbone segment, additional feature extraction layers were incorporated to harvest richer and more detailed feature information. In the neck segment, the feature fusion and upsampling strategies were adjusted to align with the requirements of the 10 × 10 detection head. Specifically, the refined feature fusion strategy ensures effective integration of features from different levels, enhancing the model’s capability to detect large-scale targets. Additionally, the introduction of an upsampling step guarantees that the information restored from deep features provides adequate spatial resolution for the 10 × 10 detection layer, thereby maintaining precise identification of large targets without compromising detection performance. To minimize the computational load, reduce noise from shallow features, and sharpen the focus on recognizing large targets, the 80 × 80 shallow detection head in the original network was removed. This redesigned network structure not only optimizes the recognition performance of large logistic parcels but also enhances the system’s robustness and applicability in complex environments.

#### FasterNetX

When enhancing the YOLOv5 network model for the task of logistics parcel identification, the addition of a new deep detection head, although beneficial for identifying large parcels, correspondingly increases the computational load. To address this challenge, this paper introduces an innovative convolution operation—PConv^27^ convolution. This method is designed to reduce computational complexity while enhancing efficiency.

PConv convolution leverages channel similarity and redundancy in feature maps to optimize computational costs. Unlike traditional convolution, PConv performs precise feature extraction on specific input channels while leaving the remaining channels unchanged, thereby reducing unnecessary computations. This approach is particularly effective for addressing issues like partial occlusion or damage, which are common in logistics parcel identification—such as partially missing parcel labels. By adeptly learning the information from surrounding pixels, PConv can reconstruct missing parts of the data, significantly enhancing recognition accuracy.

The operational efficiency of PConv markedly surpasses that of standard convolution. In terms of specific floating-point operation calculations, the computational load of PConv can be approximated as follows:

4 $$h \times w \times k^{2} \times c_{p}^{2}$$

where $h$ is the height of the feature map, $w$ is its width, $k$ is the size of the convolution kernel, and $c_{p}$ is the number of output channels.At a typical 1:4 ratio, PConv's floating-point operation count is 1/16 of a normal Conv's. Additionally, PConv has a smaller memory access volume, only 1/4 of regular convolutions:

5 $$h \times w \times 2c_{p} + k^{2} \times c_{p}^{2} \approx h \times w \times 2c_{p}$$

**Figure 9:**
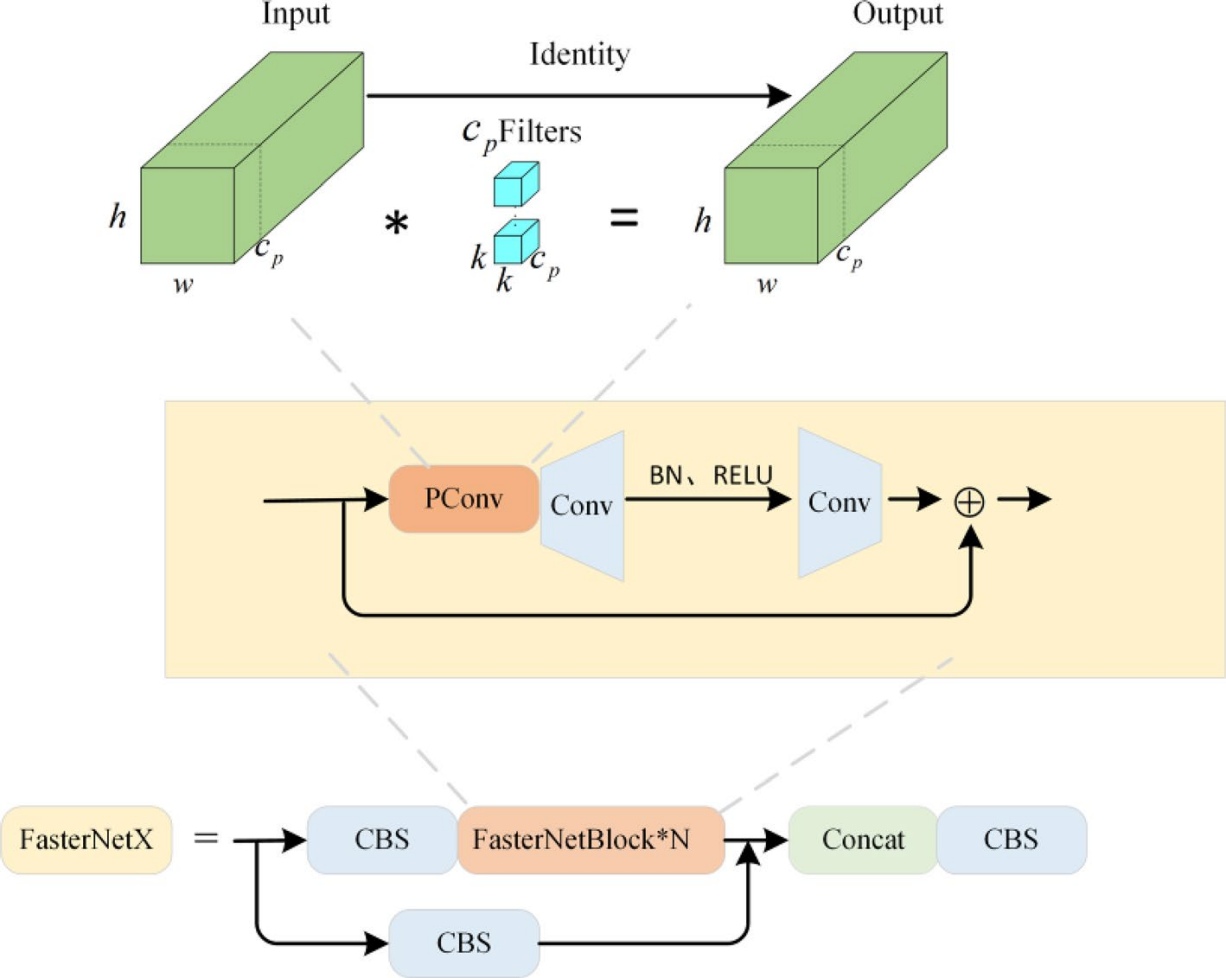
FasterNetX structure.

By integrating PConv convolution with regular convolution in the YOLOv5 network model, we effectively balance the model’s performance with computational efficiency. This integration not only enhances the processing capabilities in complex logistics scenarios but also significantly alleviates the burden on hardware resources. Figure 9 illustrates the structure of FasterNetX, distinctly marking the position of the PConv convolutional layer and highlighting its crucial role within the network.

#### ECIoU

In logistics parcel recognition, accurately determining the bounding box of a parcel is crucial. The YOLOv5 detection model currently employs the CIoU loss function, which optimizes the predicted bounding boxes by comprehensively considering three geometric factors: overlapping area, center point distance, and aspect ratio. The expression for the CIoU loss function is as follows:

6 $$L_{CIOU} = 1 - IOU + \frac{{\rho^{2} (b,b^{gt} )}}{{c^{2} }} + \alpha v$$

7 $$\alpha = \frac{v}{1 - IOU - v}$$

8 $$v = \frac{4}{{\pi^{2} }}(\arctan \frac{{w^{gt} }}{{h^{gt} }} - \arctan \frac{w}{h})^{2}$$

where $\rho (b,b^{gt} )$ represents the Euclidean distance between the center points of the predicted and actual boxes,$c$ is the diagonal distance of the smallest closed area containing both boxes, $\alpha$ is the weight coefficient, and $v$ measures the consistency between the aspect ratios of the predicted and actual boxes. $w^{gt}$ and $h^{gt}$ are the width and height of the actual box, while $w$ and $h$ are those of the predicted box.

Although CIoU performs well in addressing differences in aspect ratio and center point for object detection, its sensitivity may be insufficient for objects with extreme aspect ratios. This insensitivity can result in performance degradation when handling the diverse shapes of logistics parcels. To overcome this limitation and enhance the model’s adaptability to various parcel shapes and sizes, we have merged the advantages of CIoU and EIoU, introducing the ECIoU loss function^28^:

9 $$L_{ECIOU} = 1 - IOU + \alpha v + \frac{{\rho^{2} (b,b^{gt} )}}{{c^{2} }} + \frac{{\rho^{2} (h,h^{gt} )}}{{c_{h}^{2} }} + \frac{{\rho^{2} (h,h^{gt} )}}{{c_{w}^{2} }}$$

The ECIoU loss function not only incorporates the considerations of CIoU but also introduces additional elements to optimize handling of extreme cases. It fine-tunes the sensitivity to changes in aspect ratio and integrates additional regularization terms to better adapt to irregular shapes. This design allows ECIoU to more comprehensively evaluate the disparities between the predicted and actual bounding boxes in logistics parcel scenarios, thereby achieving more precise bounding box positioning.

By implementing the ECIoU loss function, our model demonstrates enhanced robustness and accuracy in managing diverse sizes and shapes of logistics parcels, significantly improving detection performance across various parcel types.

## Experiments

### Model training environment

All experiments were carried out in our laboratory utilizing an Intel(R) Xeon Gold 6248R CPU (3 GHz, 128 GB RAM) and NVIDIA RTX A6000 (48 GB video memory). Model training and testing took place within the PyTorch framework. Both training and testing processes employed GPU acceleration. The experimental environment utilized in this study is depicted in Table 3. Table 4 presents the training parameters for the parcel detection model.

**Table 3:** Experimental conditions.

Experimental environment	Details
Operating system	Windows 10
Translater	Pycharm
Programming language	Python3.8
Deep learning framework	Pytorch 2.0.1
CUDA version	11.8

**Table 4:** Training parameters for parcel detection model.

Training parameters	Details
Epoch	200
BatchSize	32
Image Size	640*640
workers	32

### Model evaluation metrics

To evaluate the performance of the model, we assessed several common metrics: Precision (P), Recall (R), Average Precision (AP), F1 Score (F1), and Mean Average Precision (mAP) for model performance evaluation, which are defined as follows:

10 $$precision = \frac{TP}{{TP + FP}}FN$$

11 $$recall = \frac{TP}{{TP + FN}}$$

12 $$F1 = \frac{2 \cdot precision \cdot recall}{{precision + recall}}$$

13 $$AP = \int_{0}^{1} {p(r)dr}$$

14 $$mAP = \frac{{\sum\nolimits_{i = 1}^{N} {AP_{i} } }}{N}$$

In this context, $TP$ denotes correctly predicted positive samples, $FP$ signifies incorrectly predicted negative samples, $FN$ indicates incorrectly predicted positive samples, and $N$ represents the total number of sample categories.

### Ablation study

To determine whether the improvements proposed in this study boosted model performance, an ablation study was carried out using YOLOv5n as the baseline. The test set served as the overall sample to evaluate the specific impact of these improvements on model performance.

The results of the ablation study, presented in Table 5, reveal that using an improved network structure (INS), aimed at capturing deep semantic information, notably enhanced the mAP metric. However, the incorporation of additional model parameters led to an increase in computational costs. The implementation of the PConv-designed FasterNetX module modestly improved the mAP metric while reducing the model's complexity and boosting FPS values. Additionally, ECIoU further enhanced the mAP and recall metrics upon replacing the CIOU loss function^29^. Results from Experiment 5 demonstrate that the combination of the improved network structure with the FasterNetX module increased detection speed while preserving mAP. Additionally, Experiment 6, integrating both with ECIoU, exhibited significant improvements in mAP, P, and R metrics without a marked decrease in FPS values. Table 6’s data on the detection performance of different types of parcels also indicates that more complex woven and foam parcels benefitted from greater performance improvements, thereby validating our hypothesis.

**Table 5:** Ablation experiment.

Experiment	INS	FasterNetX	ECIOU	mAP@0.5:0.95	P	R	FPS
1	–	–	–	92.5	97.9	89.4	84.7
2	√	–	–	97.7	99.4	97.4	80.2
3	–	√	–	93.7	98.6	92.1	**86.5**
4	–	–	√	94	96.5	93.5	84.7
5	√	√	–	97.5	98.3	96.9	81.8
6	√	√	√	**98.2**	**99.5**	**97.6**	81.8

Significant values are in bold.

**Table 6:** Detection performance of different parcels.

Parcel type		Bubble	Foam	Box	Paper	Plastic	Woven
Before improvement	P	97.5	96.3	98.8	98.6	96.3	100
	R	96	86.7	98.8	94.4	92.8	67.9
After improvement	P	99.3	100	99	100	98.5	100
	R	98	93.7	99.7	97.6	99.6	96.8

**Figure 10:**
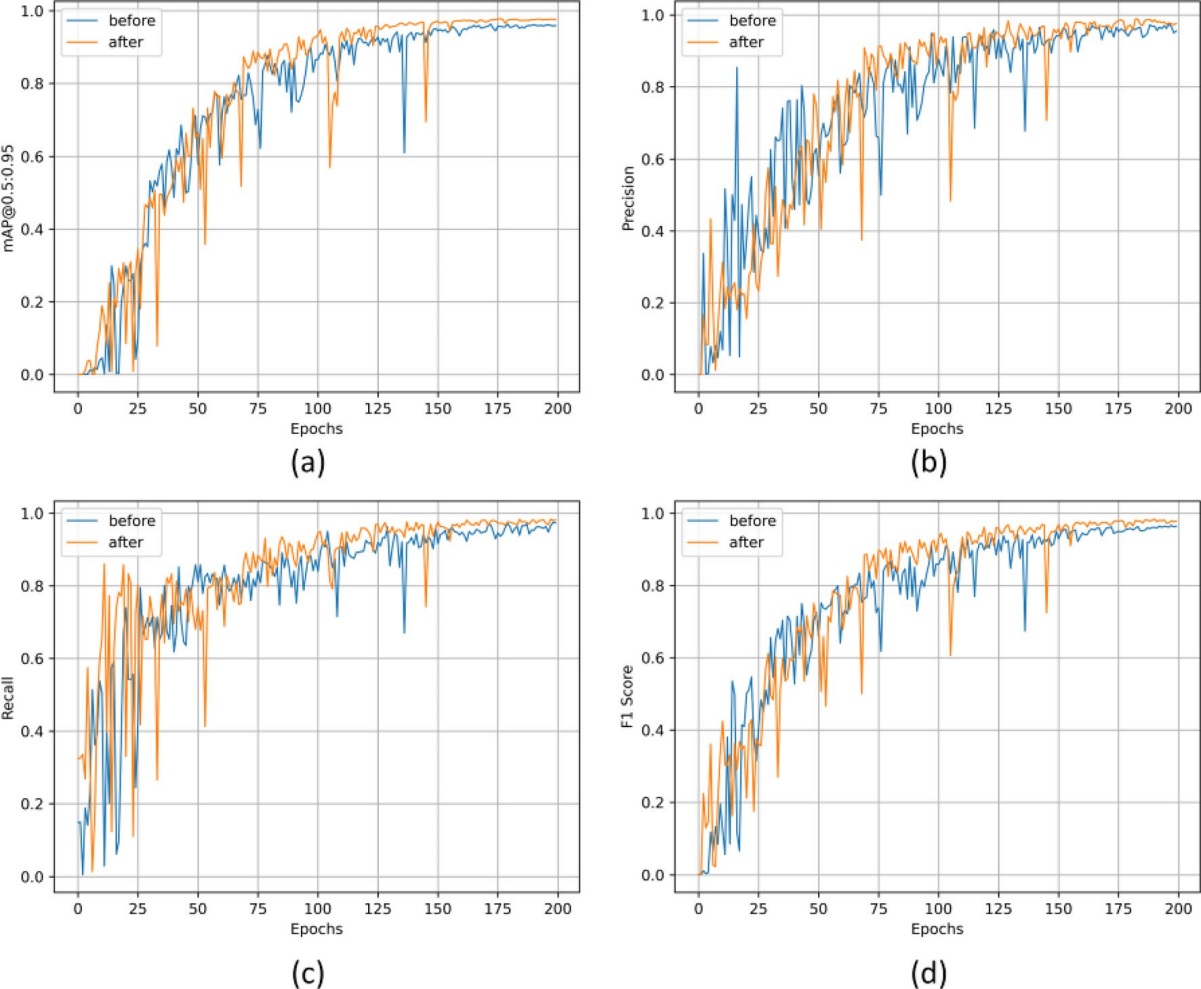
Comparison before and after the algorithm improvement. (**a**) mAP@0.5:0.95-epochs curve. (**b**) Precision-epochs curve. (**c**) Recall-epochs curve. (**d**) F1-epochs curve.

Furthermore, the training process was documented both before and after the model improvement. In Fig. 10, the blue curve depicts the model performance before improvement, while the orange curve illustrates the performance after improvement. It is evident that, when compared with the pre-improved YOLOv5n model, the curve of the improved model ascends more smoothly and converges more rapidly, demonstrating more accurate and reliable performance across all measurement indicators.

### Comparison analysis with common models

**Figure 11:**
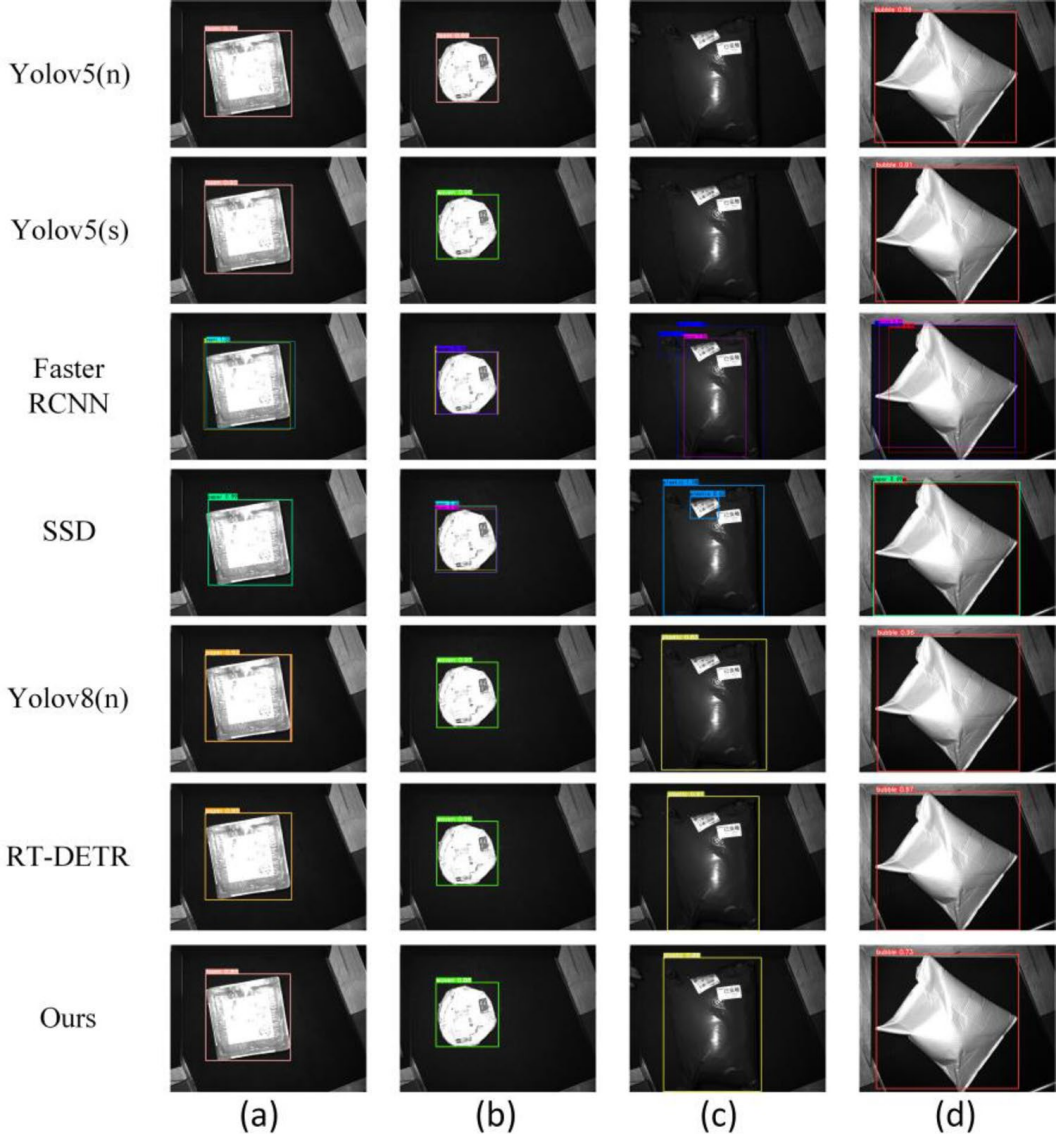
Comparison of recognition results of different models.

To further illustrate the advantages of our model, a comparative analysis was undertaken with common models. The analysis of test results from Table 7 and recognition results from Fig. 11 reveals that the YOLOv5(n) model exhibits a higher precision rate but a lower recall rate, yielding an F1 score of only 93.46%. Despite this, it excels in frame rate, demonstrating its capability for rapid image processing in real-time applications. The YOLOv5(s) model displays a significant improvement in precision and recall relative to YOLOv5(n), but with a conspicuous decrease in frame rate. Faster RCNN^30^ achieved high levels of performance in recall and F1 score but experienced a lower frame rate, attributed to its more complex architecture resulting in lower computational efficiency. SSD^31^ demonstrated strong performance in recall and F1 score but had an inferior frame rate compared to YOLOv5(n), suggesting a trade-off between high performance and speed. Compared to YOLOv5, YOLOv8 demonstrates a significant improvement in recall rate, though the enhancement in precision is less pronounced. While RT-DETR^32^ achieves high accuracy on the logistics parcel dataset, its reliance on the Transformer model introduces a complex self-attention mechanism during image processing. This mechanism typically demands greater computational resources than traditional CNNs, resulting in a substantial disadvantage in FPS. Our model excelled across all performance metrics, achieving the highest precision and F1 scores, while simultaneously maintaining a very high frame rate. This indicates that our model guarantees high accuracy while also sustaining high speed, rendering it ideal for scenarios that require high-speed logistics parcel feeding.

**Table 7:** Comparison of common model test results.

Model	P(%)	R(%)	F1(%)	FPS
yolov5(n)	97.9	89.4	93.46	84.7
yolov5(s)	98.9	93.7	96.23	74.2
Faster RCNN	94.56	**99.03**	96.74	25
SSD	96.24	98.13	97.18	37
yolov8	97.9	98.2	98.1	55
RT-DETR	98.6	97.2	97.89	15
Ours	**99.5**	97.6	**98.54**	81.8

Significant values are in bold.

### Comparison experiment of different loss functions

**Figure 12:**
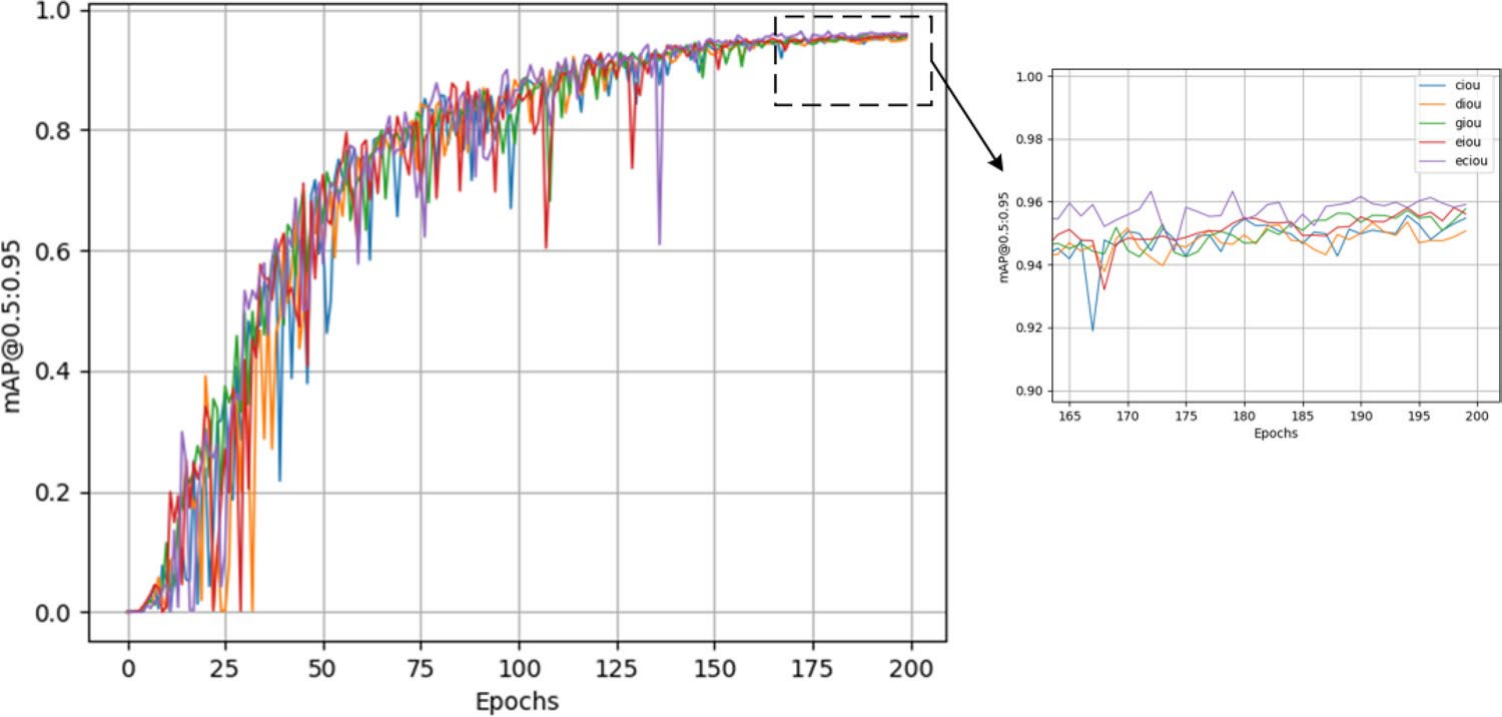
Training process diagram of different loss functions.

To confirm the enhanced detection performance of the ECIoU loss function employed in this study for logistics parcel detection scenarios, we documented the training processes of various loss functions: CIoU, DIoU^33^, GIoU^34^, EIoU^35^, and ECIoU, as illustrated in Fig. 12. In the figure, the blue curve depicts the CIoU loss function, the orange curve illustrates DIoU, the green curve shows GIoU, the red curve indicates EIoU, and the purple curve symbolizes ECIoU.

Analyzing the training process graphs of these five distinct loss functions, it becomes evident that the ECIoU loss function used in this study demonstrates the quickest convergence speed and superior detection accuracy, making it exceptionally well-suited for logistics parcel detection scenarios.

### Supplementary experiments

In the logistics industry, the parcel error rate is defined as the probability of incorrectly sorting a parcel to the wrong destination grid, serving as one of the most critical performance metrics for a cross-belt sorter. Factors such as the accuracy of parcel location detection, human interference, and assessments of parcel friction coefficients significantly influence this parcel error rate. Additionally, the average parcel supply time denotes the duration required to complete the supply task for a single parcel. A lower parcel error rate and shorter average supply time for the cross-belt sorter lead to greater economic benefits for the logistics outlets. To showcase the advantages of the intelligent classification and positioning algorithm for logistics parcels proposed in this paper, we maintained consistency in other system variables and conducted tests on positioning using light curtains, a monocular camera without traditional visual filtering, and a monocular camera with traditional visual filtering. Among the three methods, the latter two employ deep learning-based positioning technology. Each scenario involved testing 10,000 parcels. The experimental results are presented in Table 8.

**Table 8:** Comparison of different parcel supply methods.

Methods	Parcel error Rate(‰)	Average parcel supply time(s)
Positioning using light curtains	18.7	1.82
A monocular camera without traditional visual filtering	11.4	1.15
A monocular camera with traditional visual filtering	0.8	1.20

The experimental results demonstrate that the intelligent classification and positioning algorithm for logistics parcels, as proposed in this paper, significantly reduces both the parcel error rate and average parcel supply time compared to the traditional light curtain positioning algorithm. Additionally, the incorporation of traditional visual filtering into the algorithm further reduces the parcel error rate without impacting the average parcel supply time.

## Conclusion

Addressing the limitations of traditional light curtain positioning methods in logistics sorting workshops regarding high-speed upgrades, this paper introduces a logistics parcel intelligent classification and location technology utilizing a monocular camera. This technology employs traditional visual algorithms, like the average brightness value for hand filtering and the average hash algorithm for stationary parcel judgment, in conjunction with an enhanced lightweight YOLOv5 target detection algorithm, to attain high-speed and high-precision parcel positioning. This approach simplifies parcel positioning and identification, fulfills the acceleration distance requirements for ultra-high-speed parcel feeding, addresses the challenges of traditional methods in classifying parcels, and offers benefits like low cost, ease of maintenance, and straightforward industrialization. Consequently, it lays the foundation for industrial applications in high-speed logistics parcel feeding.

Taking cost into consideration, we opt for a monocular camera for positioning, which generally meets the accuracy requirements for high-speed parcel feeding in typical scenarios. However, in scenarios with more stringent parcel feeding requirements, some positioning errors may arise due to the inability to determine the height of parcels. Consequently, in practical applications, the pre-sorting of taller parcels is essential, and certain limitations remain.

Future efforts will concentrate on enhancing the system's positioning efficiency and accuracy in two primary areas. From a hardware standpoint, we plan to select industrial computers with faster processing speeds to further enhance parcel feeding speed, and contemplate the use of binocular cameras for parcel height measurement to minimize positioning errors. Conversely, from an algorithmic viewpoint, our goal is to employ more lightweight algorithms without compromising positioning accuracy and to explore techniques for estimating parcel height using a monocular camera to further decrease implementation costs.

## Data Availability

The data gathered in the experimental work of this study, supporting the findings of this work are available from the corresponding author upon reasonable request.
